# A HALDILI

**Published:** 2010

**Authors:** Rachel Hajar

## To Cure a Broken Heart

The *Jade Pendant* of the Mughal Emperor Shah Jahan is one of the prized collections of the Museum of Islamic Art in Doha, Qatar. The pendant is called a *haldili*, which was worn to cure the wearer of palpitations such as those caused by grief. The pendant, carved from pure white jade, has finely incised inscriptions consisting mainly of verses from the Qur’an as well as detail of the full titles of Shah Jahan and the date AH 1041 [1631]. Shah Jahan was heartbroken at the loss of his beloved wife, Mumtaz Mahal who died in childbirth in June of that year. The shah wore the *haldili* to help ease his grief at the loss of his wife. He built the Taj Mahal in Agra, India, in her memory.

This particular amulet is evocative of the universality of human emotions. Amulets, charms, and talismans have been worn since ancient times as protection against evil such as disease or witchcraft. They are made of wood and stone, clay, metal, plants and dead animals. They are carved into crude shapes and in the most exquisite forms. They are also comprised entirely of words, which are believed to have power and magical properties. Amulets and charms have been used by pagans, Christians, Jews, Muslims and followers of every faith and tradition known across the world. Some are considered direct links to the gods, others to local spirits. All are links to the supernatural. Regardless if they are called amulets, charms or talismans, these objects are credited with cures, health and prosperity. Amulets continue to be an important part of our modern culture.

**Figure:**
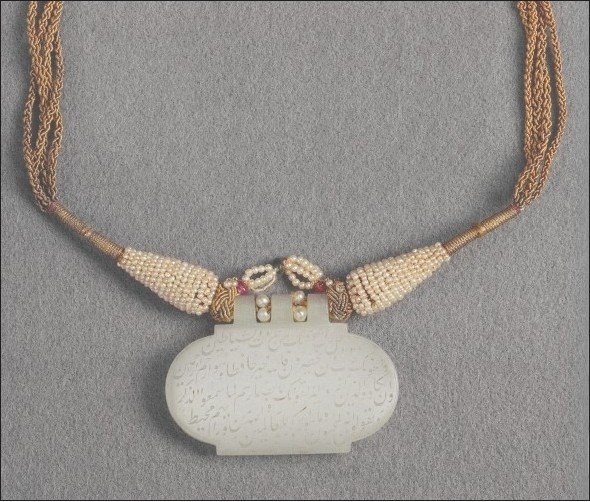
**Shah Jahan’s Jade Pendant** Museum Islamic Art, Doha, Qatar Dated: AH 1041 (1632 AD), India

